# Control of microbial growth and lipid oxidation in beef using a *Lepidium perfoliatum* seed mucilage edible coating incorporated with chicory essential oil

**DOI:** 10.1002/fsn3.2186

**Published:** 2021-04-05

**Authors:** Behrooz Alizadeh Behbahani, Fereshteh Falah, Alireza Vasiee, Farideh Tabatabaee Yazdi

**Affiliations:** ^1^ Department of Food Science and Technology Faculty of Animal Science and Food Technology Agricultural Sciences and Natural Resources University of Khuzestan Mollasani Iran; ^2^ Department of Food Science and Technology Faculty of Agriculture Ferdowsi University of Mashhad Mashhad Iran

**Keywords:** chicory essential oil, edible coating, fresh beef, *Lepidium perfoliatum* mucilage, shelf life

## Abstract

In this study, chicory essential oil (CEO) was obtained by hydrodistillation‐based extraction method and it was rich in camphor (31.3%) and phenolic compounds with outstanding antioxidant and antimicrobial properties. The CEO was then incorporated into *Lepidium perfoliatum* seed mucilage (LPSM) based aqueous solution to prepare an active CEO‐loaded LPSM edible coating. The effect of the edible coating was then investigated on the quality and shelf life of beef slices during 7 days storage at 4°C. The results revealed that beef slice coated with CEO‐loaded LPSM edible coating had a significant inhibitory effect on its lipid oxidation and microbial growth. The CEO‐LPSM coating also inhibited the weight and texture losses of beef slices during display more efficiently compared with the control and CEO‐free LPSM coating. Besides, the beef slices coated with CEO‐LPSM were the preferred samples in terms of sensory scores throughout the storage. Thus, using CEO‐rich LPSM edible coating might inhibit decay and significantly improve the shelf life of fresh beef.

## Graphical abstract

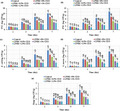

## 1 INTRODUCTION

Meat and meat products, due to their suitable pH, fermentable carbohydrate, and high contents of nitrogen, moisture, and fat, are highly prone to chemical and microbial deteriorations, which could affect the texture, flavor, color, and nutritional quality of the related products (Alizadeh Behbahani, Noshad, et al., 2020). Currently, hydrocolloid‐based edible coatings are receiving a great deal of research and industrial attention as novel food packaging systems to ameliorate shelf life and quality of food products, through preventing chemical, microbial, and physical damages (Barzegar et al., 2020). Recently, the demand for polysaccharide gums of natural origin for the development of novel edible coatings has been increased.

*Lepidium perfoliatum* (locally called Qodume shahri) is native to Iran, Iraq, Egypt, Arabia, and Pakistan, and its seed mucilage is widely used in traditional medicine to treat whooping cough, dry cough, and lung infections and as a demulcent (Koocheki et al., 2013). It has been reported that the mucilage extracted from *L. perfoliatum* seeds could be used as a stabilizing and thickening agent in food systems for viscosity increment, texture modification, and consistency stabilization purposes (Hesarinejad et al., 2014). It is also noteworthy that the *L. perfoliatum* seed mucilage (LPSM) films have good physicochemical, mechanical, and thermal properties and could be applied as a biodegradable packaging for improving the shelf life of various food products (Seyedi et al., 2015). Nonetheless, edible coatings based on polysaccharides have hardly antioxidant and antimicrobial activity themselves; therefore, food‐grade ingredients (e.g., plant‐based antioxidant and antimicrobial components) are commonly added to edible coatings to improve their biological and functional properties (Barzegar et al., 2020).

In this context, essential oils (EOs) are frequently used in food preservation technologies as natural and are generally recognized as safe agents, owing to antimicrobial and antioxidant compounds they carry (Calo et al., 2015; Rehman et al., 2020). For example, it has been reported that the lemon/thyme EOs‐enriched chitosan coating maintained higher hardness and color and retarded lipid/protein oxidation and microbial growth in grass carp filled during cold storage (Cai et al., 2018). Similarly, Cai et al. (2015) demonstrated that the fresh fish fillets treated with clove, cumin, and spearmint oils maintained the hardness, delayed protein and nucleotide degradation, retarded the sensory deterioration, and inhibited the microbial growth and the formation of biogenic amines.

Chicory (*Cichorium intybus* L.) is a perennial herb and rich in bioactive compounds for human food fortification purposes. It also has many types of biological properties, such as anticancer, antidiabetic, anti‐inflammatory, hepatoprotective, hypolipidemic, antioxidant, and antimicrobial effects (Šaponjac et al., 2021). Moreover, the chicory essential oil (CEO) has remarkable antioxidant and antimicrobial properties (Gol et al., 2014). Accordingly, CEO could be used as a potential natural preservative in edible coatings to amend their oxidation‐ and microbial growth‐suppression functions.

To the best of our knowledge, there is no evidence about the role of the oxidative and microbial stability of meat and meat products wrapped by CEO‐loaded LPSM edible coating. The objective of this study was therefore to isolate and characterize the CEO and develop a novel CEO‐loaded LPSM edible coating to improve the microbial and oxidative stability of beef slices during cold storage.

## 2 MATERIALS AND METHODS

### 2.1 Materials

The *L. perfoliatum* seeds and chicory were purchased from a local market (Khuzestan, Iran). β‐carotene, Linoleic acid, ABTS (2,2′‐Azino‐bis (3‐ethylbenzothiazoline‐6‐sulfonic acid) diammonium salt), quercetin, gallic acid, and DPPH (2,2‐diphenyl‐1‐picrylhydrazyl) were purchased from Sigma‐Aldrich Co. Mannitol Salt Agar (MSA), Eosin Methylene Blue (EMB), Mueller Hinton Broth (MHB), Mueller Hinton Agar (MHA), Sabouraud Dextrose Broth (SDB), Sabouraud Dextrose Agar (SDA), and Plate Count Agar (PCA) were obtained from Merck Co.

### 2.2 CEO extraction

The CEO extraction was performed according to the hydrodistillation method, which is known as one of the famous, routine, and official standard procedures to extract and control the quality of EOs of medicinal herbs (Taherpour et al., 2017). For the extraction of CEO, the chicory was dried, powdered, and added to the Clevenger device (containing 50 g powder in 750 ml distilled water). The extraction process was performed for 3 hr at 335 power, according to a method reported by Heydari et al. (2020), with some changes. The oil (CEO) was then collected, dehydrated, and stored at 4°C.

### 2.3 Gas chromatography‐mass spectroscopy (GC/MS)

A gas chromatograph (GC; Agilent 7890A) coupled to a mass spectrometer (MS; Agilent 5975C) was employed for the identification and quantification of the main chemical compounds of the CEO. Briefly, 0.2 µl of the oil was injected to the DB‐5 capillary column (30 m × 0.25 mm × 0.25 µm) and the heating rate, ionization energy, helium gas flow rate was set at 5°C/min, 70 eV, and 1 ml/min, respectively. Finally, the retention profiles were obtained and compared with those of know samples which were analyzed under the same conditions (Alizadeh Behbahani & Shahidi, 2019).

### 2.4 Total phenolics and flavonoids content

Total phenolic content of the CEO was measured based on the method described by Ahmed et al. (2019). Gallic acid (0–0.5 mg/ml) was used as a standard to obtain a calibration curve and the total phenolic content of the oil was expressed as mg gallic acid equivalent (GAE)/g.

The procedure of Saki et al. (2019) was used to determine the total flavonoid content of the CEO. Briefly, the absorbance of the mixture of CEO, NaNO_2_, AlCl_3_, NaOH, and distilled water was recorded at 510 nm, and the content of total flavonoids of the CEO was then expressed as mg quercetin equivalent (QE)/g.

### 2.5 Antioxidant activity

In this study, DPPH‐radical scavenging (DPPH‐RS) activity, ABTS radical scavenging (ABTS‐RS) activity, and β‐carotene‐linoleic acid bleaching assays were used to determine the antioxidant activity of the oil, according to the method described by Barzegar et al. (2020).

To determine the DPPH‐RS activity, the CEO (50 μl) or control (50 50 μl) was mixed with 0.12 mM ethanolic DPPH solution (5 ml). The resulting solution was stored at 25°C for 30 min, and its absorbance (*A*) was read at 517 nm. The DPPH‐RS activity was then measured as below:

$$\text{DPPH}‐\text{RS activity}\;\left(\%\right)=\frac{A_{\text{control}}‐A_{\text{CEO}}}{A_{\text{control}}}\times 100.$$

For the ABTS‐RS activity, ABTS solution and K_2_S_2_O_8_ were initially mixed together to generate ABTS radical cation solution. After that, the CEO (0.1 ml) or control (0.1 ml) was mixed with the ABTS radical solution (3.9 ml) and its absorbance was recorded at 734 nm. The ABTS‐RS activity was then measured as follows:

$$\text{ABTS}‐\text{RS activity}\;\left(\%\right)=\frac{A_{\text{control}}‐A_{\text{CEO}}}{A_{\text{control}}}\times 100.$$

The following equation was used to measure the inhibitory effect of the CEO against β‐carotene‐linoleate solution bleaching:

$$\text{Inhibitory effect}\;\left(\%\right)=\frac{A_{\text{S(120)}}‐A_{\text{C(120)}}}{A_{\text{C(0)}}‐A_{\text{C(120)}}}\times 100,$$

where, *A*_S(120)_ is the absorbance of the solution at 490 nm after 120 min incubation, *A*_C(0)_ is the absorbance of control at the time zero, and *A*_C(0)_ is the absorbance of control at the after 120 min reaction.

### 2.6 Antimicrobial activity

The method of Noshad et al. (2018) was used to evaluate the antibacterial effect of the CEO against some pathogenic gram‐positive bacteria (*Staphylococcus aureus*, *Listeria innocua*, and *Bacillus cereus*) and gram‐negative bacteria (*Escherichia coli*, *Pseudomonas aeruginosa*, and *Salmonella typhi*), through disk diffusion agar (DDA), well diffusion agar (WDA), and minimum inhibitory/bactericidal concentration (MIC/MBC) methods.

### 2.7 Mucilage extraction

The *L. perfoliatum* seed mucilage (LPSM) was extracted according to the method of Koocheki et al. (2013). The seeds were dispersed in deionized water (1:30 ratio), and the extraction process was performed for 90 min at pH 8 and 48°C. The slurry was oven‐dried (45°C), milled, sieved, and stored at 4°C.

### 2.8 Beef slices coating

The edible coatings were prepared by mixing LPSM (2 g) and Tween‐80 (1 ml) in distilled water (the volume was made up to 100 ml). The CEO (0, 0.5, 1, and 1.5% v/v) was then added. The beef slices were immersed in the CEO‐LPSM solution for 60 s and then air‐dried for 10 min. The essential oil concentrations and coating treatments were selected according to the preliminary experiments for the beef slices to assure (a) adherence and steadiness of the LPSM coating, (b) antimicrobial and antioxidant activity of the essential oil, and (c) acceptable sensory properties (masking the strong flavor or odor of the essential oil). The coated beef slices were then stored at 4°C for 7 days. The noncoated beef slice was used as control. The samples were designated as control, LPSM + 0%CEO, LPSM + 0.5%CEO, LPSM + 1%CEO, and LPSM + 1.5%CEO.

### 2.9 Microbial analysis

To determine the microbial load changes of beef slices during display, the slices were firstly mixed and homogenized with 0.1% peptone water in a Stomacher. The slurry was then added to the test tubes containing 0.1% peptone water to prepare subsequent dilutions (10^–1^ to 10^–6^), which were further inoculated into the plates containing culture medium. The following tests were then performed to evaluate the microbial growth during storage (Alizadeh Behbahani, Noshad, et al., 2020):

● Total viable count (TVC) bacterial count in PCA (48 hr incubation at 37°C)
● Psychrotrophic count (PTC) in PCA (10 days incubation at 7°C)
● *Staphylococcus aureus* count in MSA (24 hr incubation at 37°C)
● *Escherichia coli* count in EMB (24 hr incubation at 37°C)
● Fungi count in SDA (72 hr incubation at 27°C).

### 2.10 Physicochemical analysis

#### 2.10.1 Lipid oxidation

The peroxide value (PV; meq O_2_/kg) and thiobarbituric acid (TBA; mg MDA/kg) value of beef slices were measured according to the methods of Bazargani‐Gilani et al. (2015).

#### 2.10.2 Moisture content

The beef slices were dried at 105°C for 3 hr, and the moisture content was then determined (AOAC, 1995).

#### 2.10.3 pH measurement

The beef samples (10 g) were mixed with deionized water (90 ml) and homogenized (30 s, 13,000 rpm, 25°C). The pH value of the slurry was then determined by a pH‐meter (Barzegar et al., 2020).

#### 2.10.4 Hardness

A cylindrical probe was used to compress the beef slices (30% of the sample thickness) at a constant pretest speed, test speed, and post‐test speed of 3, 1, and 3 mm/s, respectively, by a Stable Micro System Texture Analyzer (TA, XT2i). The highest force (N) was reported as the hardness of the beef slices.

#### 2.10.5 Sensory evaluation

The odor, color, texture, and overall acceptance of the coated and control beef slices were evaluated by 25‐well trained panelists via a nine‐point hedonic scale test. The sensory scores ranked from best (9 = like extremely) to worst (1 = dislike extremely).

#### 2.10.6 Statistical analysis

Data were analyzed by Minitab software (version 16) via one‐way ANOVA. The Tukey test, at confidence level of 95% (*p* < .05), was applied to determine the differences between the data means. It is also necessary to note that the experiments were done at three replications.

## 3 RESULTS AND DISCUSSION

### 3.1 CEO characterization

Plant EOs are rich in bioactive compounds with versatile biological functions. In this study, CEO was subjected to GC/MS apparatus to identify and quantify its main chemical constituents. The oil was rich in camphor (31.3%), cymene (18.2%), γ‐terpinene (8.45%), and β‐pinene (7.85%). Other compounds, such as pinene (4.9%), eucalyptol (3.3%), camphene (3.1%), and carvacrol (2.7%), were also found in the CEO at lower concentrations. In accordance with our study, it was reported that the major components of the CEO are camphor (20.74%), cymene (15.06%), γ‐terpinene (13.24%), and cuminal (10.79%) (Gol et al., 2014). Similarly, it was found that camphor (21.4%–26%) is the main compound of the CEO (Farhoudi, 2017). However, the variation found in the concentration and type of the major constituents is due to the fact that the composition of volatile organic compounds is greatly dependent on the genetic factors, harvest time, growing location, storage conditions, nutrients, light, water, and extraction methods (Šaponjac et al., 2021).

The CEO also contained considerable levels of phenolic and flavonoid compounds (73.45 ± 0.7 mg GAE/g and 67.62 ± 0.58 mg QE/g, respectively). The content of total phenolic and flavonoid compounds is indeed dependent on the chicory culture, harvest time, other environmental factors, and extraction method/solvent. Accordingly, the cultivated and wild chicory roots have significantly different total phenolic contents of 35.1 and 22.4 mg GAE/100 g, respectively (Spina et al., 2008). Likewise, it seems that chicory leaves have noticeably higher total phenolic contents in comparison with other parts of the plant (Heimler et al., 2009). The total flavonoid content of 112.38 mg QE/100 g has been also found in chicory leaves (Khalaf et al., 2018). Indeed, chicory plant is a rich source of phenolic compounds, such as gallic acid, syringic acid, catechin, caffeic acid, vanillic acid, protocatechic acid, ferulic acid, chlorogenic acid, naringin, hesperidin, neohesperidin, myricetin, kaempferol, quercetin, rutin hydrate, and resveratrol (Sahan et al., 2017). These phenolic compounds have the potential to possess antioxidant and antimicrobial activities and improve the shelf life of food products.

Antioxidant capacity is occurred by various mechanisms, which indicates utilizing a method based on one mechanism may not demonstrate the true antioxidant activity (Karadag et al., 2009). Thus, several antioxidant tests, such as DPPH‐ and ABTS radical scavenging activity and β‐carotene‐linoleic acid model system, were used to measure the antioxidant effect of the CEO. The oil had a DPPH‐radical scavenging effect of 72.95 ± 0.53%, and this remarkably high antioxidant capacity indicates that the CEO has hydrogen/electron ability to reduce DPPH free radicals to nonreactive molecules. The oil was also able to scavenge ABTS radicals by 64.59 ± 0.4%, probably through hydrogen atom and electron transfer mechanisms. It was also observed that the CEO had a moderate inhibitory effect against β‐carotene discoloration (54.5 ± 0.47%) likely via neutralizing linoleate free radicals. The antioxidant activity of chicory extracts and EOs has been reported in the literature (Gol et al., 2014; Khalaf et al., 2018; Sahan et al., 2017). This positive biological activity of the CEO is mainly due to the redox properties of its phenolic compounds, thereby acting as hydrogen/electron donors, singlet oxygen quenchers, and metal ions chelation (Indrianingsih et al., 2015).

**FIGURE 1:**
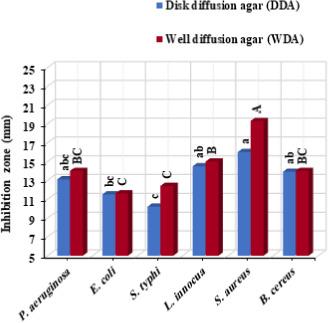
Antimicrobial effect of chicory essential oil (CEO) against some pathogenic and spoilage bacterial species

As can be seen from Figure 1 and Table 1, the CEO showed considerable antibacterial effect against all tested microorganisms. The gram‐positive bacteria (*S. aureus*, *B. cereus*, and *L. innocua*) were more susceptible to the oil compared with the gram‐negative ones (*S. typhi*, *E. coli*, and *P. aeruginosa*) as confirmed by the higher inhibition zones in the DDA (14.8 mm vs. 11.6 mm) and WDA (16.1 mm vs. 12.66 mm) tests. Moreover, it can be observed that the inhibition zones in the WDA antimicrobial assay (14.38 mm) were generally higher compared with those in the DDA test (13.2 mm). This might be due to the direct interaction between the oil and the microorganism in the WDA method; while, the diffusion of CEO from the disks surface to the medium determines its antibacterial effect in the DDA technique (Alizadeh Behbahani & Imani Fooladi, 2018a; Barzegar et al., 2020). It could be also noteworthy that low levels of CEO were generally enough to possess growth‐suppression or killing effect against gram‐positive microorganisms, in comparison with the gram‐negative ones (Table 1). The higher susceptibility of the gram‐positive bacterial species to the oil is greatly due to the presence of a thin and single mucopeptide layer in their cell membrane, whereas, the outer cell membrane of the gram‐negative ones is covered and protected by a complex lipopolysaccharide layer which could function as a barrier toward diffusion of hydrophobic antimicrobial agents across the cell (Alizadeh Behbahani, Falah, et al., 2020; Alizadeh Behbahani et al., 2019; Alizadeh Behbahani, Noshad, et al., 2020; Noshad et al., 2021; Yeganegi et al. 2018). These results are supported by the findings of Majd et al. (2009) and Khalaf et al. (2018).

**TABLE 1:** The minimum inhibitory concentration (MIC) and minimum bactericidal concentration (MBC) of the chicory essential oil (CEO) on some pathogenic microorganisms

Microorganism	MIC (mg/ml)	MBC (mg/ml)
*Pseudomonas aeruginosa*	100	400
*Escherichia coli*	200	400
*Salmonella typhi*	400	>400
*Listeria innocua*	100	200
*Staphylococcus aureus*	25	100
*Bacillus cereus*	100	200

### 3.2 Application of CEO‐loaded LPSM edible coating on beef slices

The CEO with considerable antimicrobial and antioxidant properties was therefore used as a natural preservative to develop novel active edible coatings (i.e., CEO‐loaded LPSM coating) to ameliorate quality and shelf life of beef slices.

**FIGURE 2:**
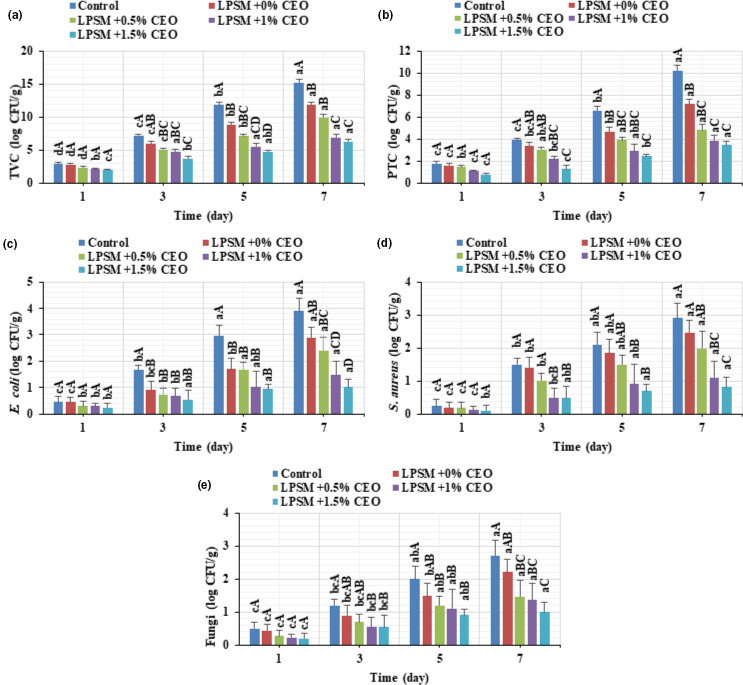
Changes in total viable count (a), psychrotrophic count (b), *Escherichia coli* count (c), *Staphylococcus aureus* count (d), and fungi count (e) of the beef slices stored at 4°C for 7 days

Figure 2 indicates the changes in TVC, PTC, *E. coli*, *S. aureus*, and fungi counts of the control and coated beef slices during cold storage. The samples experienced a significant increase in TVC as a function of storage period (Figure 2a), and the coated samples had significantly lower TVC than the noncoated one. Indeed, the TVC of the beef slices increased up to 5.24‐, 4.23‐, 4.16‐, 3.22‐, and 3.15‐fold in control, LPSM + 0%CEO, LPSM + 0.5%CEO, LPSM + 1%CEO, and LPSM + 1.5%CEO coated samples, respectively, by the end of storage time. It is also worth to note that a TVC of 7 log CFU/g is the maximum permitted TVC level for fresh beef (Alizadeh Behbahani, Noshad, et al., 2020). In this context, the beef slices exceeded the permitted level on day 3 (control) and day 5 (LPSM + 0%CEO and LPSM + 0.5%CEO), while the LPSM + 1%CEO and LPSM + 1.5%CEO coated beef samples never reached this limit level during refrigeration storage. Accordingly, the microbial shelf lives of 2, 4, 4, 7, and 7 days are predicted for the control, LPSM + 0%CEO, LPSM + 0.5%CEO, LPSM + 1%CEO, and LPSM + 1.5%CEO coated samples, respectively. This might be likely due to the antimicrobial effect of the CEO and oxygen barrier function of the CEO‐rich edible coatings, in accordance with other studies (Alizadeh Behbahani et al., 2017).

Similar trends were observed for the PTC, *E. coli*, *S. aureus*, and fungi counts in the control and coated samples (Figure 2b–e). Despite the fact that all the beef slices experienced a significant increase (*p* < .05) in the microbial growth over time, the coated samples had significantly lower microbial counts compared with the noncoated one. And this effect was more pronounced in the samples wrapped by LPSM + 1.5%CEO. The positive effect of the CEO‐loaded LPSM edible coating on the beef preservation could be mainly ascribed to (a) the antimicrobial activity of the oil and (b) the oxygen barrier characteristics of the edible coating. The growth of aerobic microorganism, such as fungi and psychrotrophic bacteria, which are the main cause of meat spoilage during cold storage under aerobic conditions, is therefore inhibited remarkably (Alizadeh Behbahani & Imani Fooladi, 2018a, 2018b). These results are in accordance with those of Kiarsi et al. (2020), who reported that EOs‐loaded seed mucilage‐based edible coatings could significantly suppress the growth of microorganisms (i.e., TVC, PTC, *E. coli*, *S. aureus*, and fungi) during cold storage.

**FIGURE 3:**
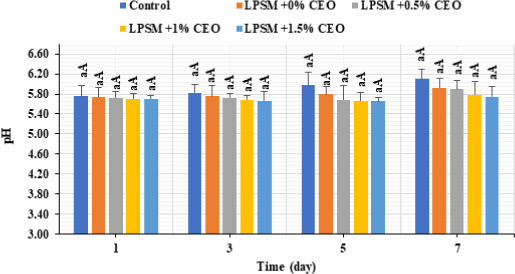
Changes in pH value of the coated and noncoated beef slices stored at 4°C for 7 days

**FIGURE 4:**
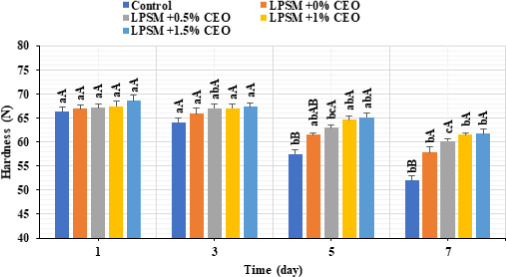
Changes in hardness value of the coated and noncoated beef slices stored at 4°C for 7 days

Accordingly, the beef slices coated by the CEO‐rich LPSM had the lowest changes in pH value (Figure 3) and hardness (Figure 4) throughout the storage time. Although all the samples experienced an increase in pH value as a function of storage time, the CEO and edible coatings had no significant effects on the pH value of the samples (*p* > .05). However, the higher CEO concentration in the LPSM edible coating, the lower were the pH increment (*p* > .05) and hardness loss (*p* < .05). The samples coated by CEO‐loaded LPSM had significantly higher hardness value compared with the control sample, at the 7th day of storage. This could probably due to microbial growth‐suppression and meat enzymes activity‐inhibitory effects of the CEO and edible coating, thereby preventing meat protein degradation and subsequent pH increase and texture loss (Mohan et al., 2012; Omidi‐Mirzaei et al., 2020).

**FIGURE 5:**
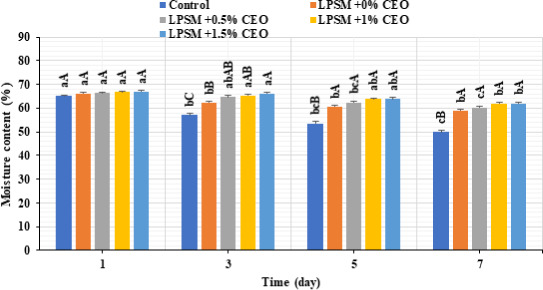
Changes in moisture content of the coated and noncoated beef slices stored at 4°C for 7 days

The edible coating was also able to efficiently inhibit weight loss of the beef slices over time (Figure 5). Despite the fact that all the samples underwent a significant decrease in moisture content (i.e., higher weight loss) during cold storage, the beef slices coated by the CEO‐enriched LPSM maintained moisture content compared with the noncoated sample. At the end of refrigeration period, the control sample underwent a significant weight loss of about 23.23%, whereas the samples coated by LPSM + 0%CEO, LPSM + 0.5%CEO, LPSM + 1%CEO, and LPSM + 1.5%CEO had lower weight losses of 10.89%, 9.24%, 7.18%, and 7.35%, respectively. The less weight loss by CEO‐loaded LPSM edible coating could be attributed to the water barrier properties of the coating (Ruan et al., 2019). In line with our study, little exudates were observed in meat samples coated by bioactive‐loaded edible coating and the meat protected against water losses during storage (Alexandre et al., 2020).

**FIGURE 6:**
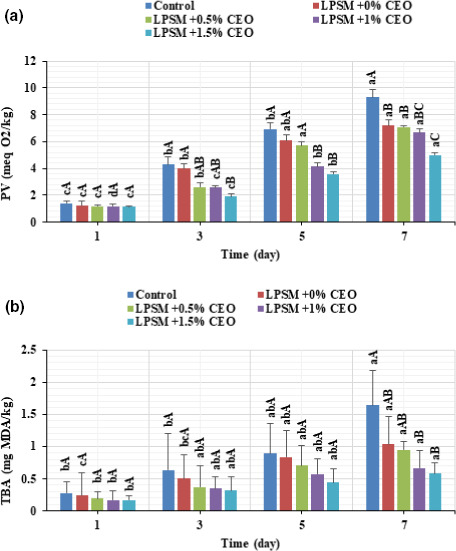
Changes in peroxide value (a) and thiobarbituric acid value (b) of the coated and noncoated beef slices stored at 4°C for 7 days

It is also necessary to point out that lipid oxidation is considered as one of the main factors of quality deterioration in beef. The PV and TBA value were then measured as lipid oxidation indices (Figure 6). As can be observed in Figure 6a, the PV increased significantly during refrigeration storage. The uncoated beef sample experienced the highest PV increase during storage (6.69‐fold). While the PV of the coated samples increased remarkably slowly, and the LPSM + 0%CEO, LPSM + 0.5%CEO, LPSM + 1%CEO, and LPSM + 1.5%CEO showed approximately 5.85‐, 5.95‐, 5.71‐, and 4.31‐fold PV increase as the storage time rose from 1 to 7 days. It is noteworthy that the permitted PV limit for meat is 7 meq O_2_/kg (Alizadeh Behbahani, Noshad, et al., 2020). Accordingly, the uncoated beef sample exceeded the permitted limit on the 7th day and its shelf life is therefore predicted to be 5 days under cold storage conditions; while, the beef samples coated by LPSM + 1%CEO and LPSM + 1.5%CEO had lower PVs than the limited value, and the shelf life of these coated meat samples is greater than 7 days.

Before storage (day 1), the TBA levels were similar in the beef slices from uncoated and CEO‐loaded LPSM coated treatments and lowest from treatments containing higher CEO concentrations (*p* > .05) (Figure 6b). As expected, the TBA values increased significantly during storage (*p* < .05), mainly in the noncoated sample, which presented the highest value of 1.64 MDA/kg at the end of storage period, greater than the TBA limit for acceptability in meat (i.e., 1 MDA/kg) (Alizadeh Behbahani & Imani Fooladi, 2018a, 2018b). However, the addition of CEO into LPSM edible coating decreased the TBA progression more efficiently, and all the CEO‐loaded LPSM coated beef slices had lower TBA levels than the acceptable limit. This study showed that the use of CEO‐rich edible coating was effective in delaying the oxidation of beef during 7 days of refrigeration display, probably due to the antioxidant activity of the oil and the ability of the edible coating to minimize the contact with oxygen and light (Alizadeh Behbahani & Imani Fooladi, 2018a, 2018b; Barzegar et al., 2020).

**FIGURE 7:**
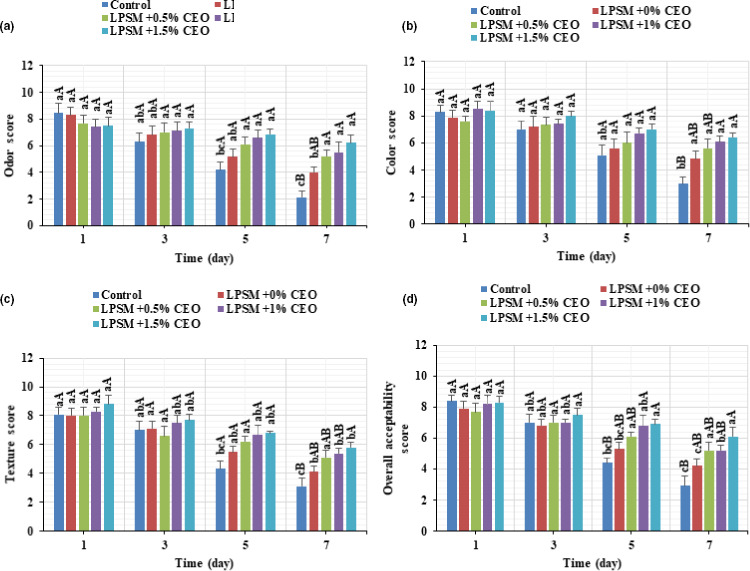
Changes in odor (a), color (b), texture (c), and overall acceptance (d) of the coated and noncoated beef slices stored at 4°C for 7 days

Oxidation and microbial growth in foods are also associated with consumer rejection. As shown in Figure 7, all sensory properties of beef samples (odor, color, texture, and overall acceptance), whether coated or not, decreased progressively as a function of storage time; however, the beef slices with CEO were the preferred beef and all treatments with edible coating received higher scores in comparison with the control. From the panelist points of view, the meat samples could be only accepted when the sensory properties received high scores, greater than 4 (Heydari et al., 2020). In this context, the control sample was unacceptable in terms of all sensory features after 7 days storage, whereas the CEO‐loaded LPSM coated beef slices were acceptable throughout the display. Indeed, the beef slices coated by CEO‐rich LPSM, which had the highest oxidative and microbial stability, ranked the highest sensory scores, as well. This is mainly due to the antimicrobial and antioxidant activity of the oil and oxygen/water barrier properties of the edible coating, inhibiting oxidation and microbial growth and subsequent quality loss of meat (Alizadeh Behbahani, Noshad, et al., 2020; Barzegar et al., 2020; Kiarsi et al., 2020; Rezaeifar et al., 2020). Moreover, the flavor and odor conferred on the beef by the CEO could also have influenced its consumer acceptance.

## 4 CONCLUSIONS

The chicory essential oil showed a large number of bioactive compounds with superb antioxidant and antimicrobial properties. The incorporation of chicory essential oil into the *L. perfoliatum* seed mucilage‐based edible coating reduced the beef lipid oxidation and microbial growth more efficiently compared with the oil‐free coating. The bioactive‐loaded edible coating also decreased weight and texture losses during display and improved beef acceptability. Therefore, edible coatings rich in natural compounds with superb antimicrobial and antioxidant effects could be used in animal meat products to ameliorate their shelf life.

## CONFLICT OF INTEREST

The authors have declared no conflict of interest.

## ETHICAL APPROVAL

This article does not contain any studies with human or animal subjects.
